# Promiscuity and specificity of eukaryotic glycosyltransferases

**DOI:** 10.1042/BST20190651

**Published:** 2020-06-15

**Authors:** Ansuman Biswas, Mukund Thattai

**Affiliations:** Simons Centre for the Study of Living Machines, National Centre for Biological Sciences, TIFR, Bangalore, India

**Keywords:** biosynthesis, glycosylation, promiscuity

## Abstract

Glycosyltransferases are a large family of enzymes responsible for covalently linking sugar monosaccharides to a variety of organic substrates. These enzymes drive the synthesis of complex oligosaccharides known as glycans, which play key roles in inter-cellular interactions across all the kingdoms of life; they also catalyze sugar attachment during the synthesis of small-molecule metabolites such as plant flavonoids. A given glycosyltransferase enzyme is typically responsible for attaching a specific donor monosaccharide, via a specific glycosidic linkage, to a specific moiety on the acceptor substrate. However these enzymes are often promiscuous, able catalyze linkages between a variety of donors and acceptors. In this review we discuss distinct classes of glycosyltransferase promiscuity, each illustrated by enzymatic examples from small-molecule or glycan synthesis. We highlight the physical causes of promiscuity, and its biochemical consequences. Structural studies of glycosyltransferases involved in glycan synthesis show that they make specific contacts with ‘recognition motifs’ that are much smaller than the full oligosaccharide substrate. There is a wide range in the sizes of glycosyltransferase recognition motifs: highly promiscuous enzymes recognize monosaccharide or disaccharide motifs across multiple oligosaccharides, while highly specific enzymes recognize large, complex motifs found on few oligosaccharides. In eukaryotes, the localization of glycosyltransferases within compartments of the Golgi apparatus may play a role in mitigating the glycan variability caused by enzyme promiscuity.

## Introduction

**Figure 1.**
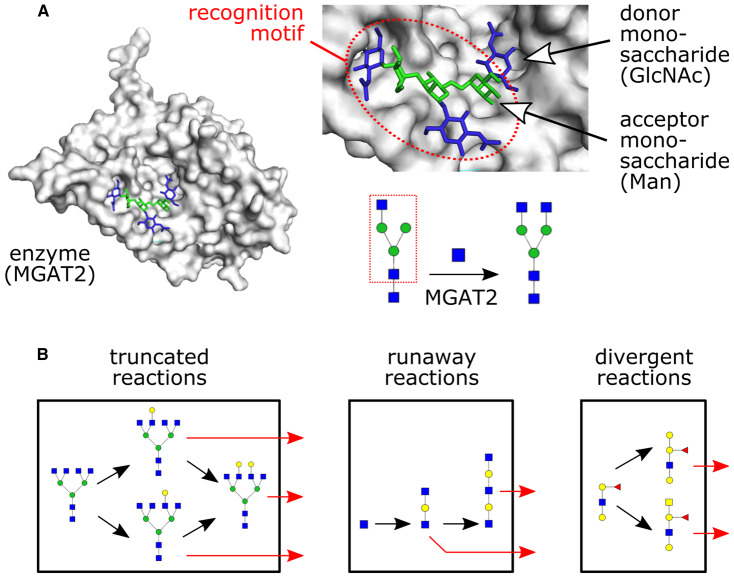
Attachment of a donor monosaccharide to an acceptor, catalyzed by a glycosyltransferase enzyme. (**A**) The enzyme MGAT2 catalyzes the attachment of a GlcNAc donor monosaccharide to a mannose acceptor monosaccharide. Black arrows show monosaccharide addition reactions. The enzyme makes specific contacts with multiple moieties of the acceptor, which we term its ‘recognition motif’ (dotted red oval/box). The protein structure is based on PDB id: 5VCS; the position of the donor monosaccharide is modeled. (**B**) In eukaryotes, glycan synthesis by glycosyltransferase enzymes takes place within membrane-bounded reaction compartments (black boxes) such as the lumen of the ER or the cisternae of the Golgi apparatus. Growing oligosaccharides can exit the reaction compartment (red arrows). We highlight the three enzymatic causes of glycan microheterogeneity: truncated, runaway and divergent reactions.

The biosynthesis of several physiologically important small molecules and oligosaccharides requires the covalent attachment of monosaccharides. This process, termed glycosylation, is carried out by specialized enzymes known as glycosyltransferases (GTases) [1]. GTases catalyze glycosidic linkages between monosaccharides on a donor substrate (usually a nucleotide-linked sugar) and a moiety on an acceptor substrate (a small molecule or oligosaccharide) (Figure 1A). Many types of organic substrates can be glycosylated. Plant secondary metabolites such as flavonoids form a diverse class of glycosylated small molecules with industrial and pharmacological applications [2,3,4]. Glycans, a particularly important class of molecules synthesized by GTases, are linear or branched oligosaccharides covalently linked to proteins or lipids. Glycans are abundant on the cell surfaces of both prokaryotes and eukaryotes, and play critical roles in inter-cellular interactions [1,5].

GTases fall into three structural classes (GT-A, GT-B, and GT-C) [6], each split into many sub-families based on sequence homology. Enzymes belonging to all three classes carry out glycan synthesis, while members of the large GT-1 sub-family of GT-B enzymes participate in small-molecule synthesis [2,6,7]. Animal and plant genomes encode hundreds of GTases [2,8,9], each responsible for linking donor monosaccharides such as glucose, galactose, mannose, fucose, sialic acid, xylose, N-acetylglucosamine (GlcNAc), N-acetylgalactosamine (GalNAc), or glucuronic acid (GlcA) to specific acceptors. This large enzymatic repertoire allows organisms to use the same set of monosaccharides to construct a large variety of small molecules and glycan oligosaccharides [4,10].

The nomenclature of GTases reflects their preferred donor monosaccharide. Thus, galactosyltransferases add galactose, fucosyltranferases add fucose, and sialyltranferases add sialic acid to the acceptor. However, these enzymes typically act on many types of acceptors, and can occasionally act on multiple types of donors or catalyze multiple types of linkages. A recent mass-spectrometric study of GT-1 GTases in *Arabidopsis thaliana* found that the majority of these enzymes were promiscuous [11]. Similarly, many glycan biosynthetic GTases are promiscuous [8]. Enzyme promiscuity can result in variability of the final biosynthetic product [12,13,14,15,16] (Figure 1B). For example, a single glycosylated protein type purified from a single cell type is typically associated with multiple glycan oligosaccharide variants [10], a phenomenon known as microheterogeneity. In eukaryotes, glycan synthesis by GTases occurs within the lumen of the ER and the compartments of the Golgi apparatus [17]. The compartmentalization of GTases can partly mitigate the consequences of enzyme promiscuity, by preventing runaway and divergent reactions that would otherwise cause glycan variability [12].

**Figure 2.**
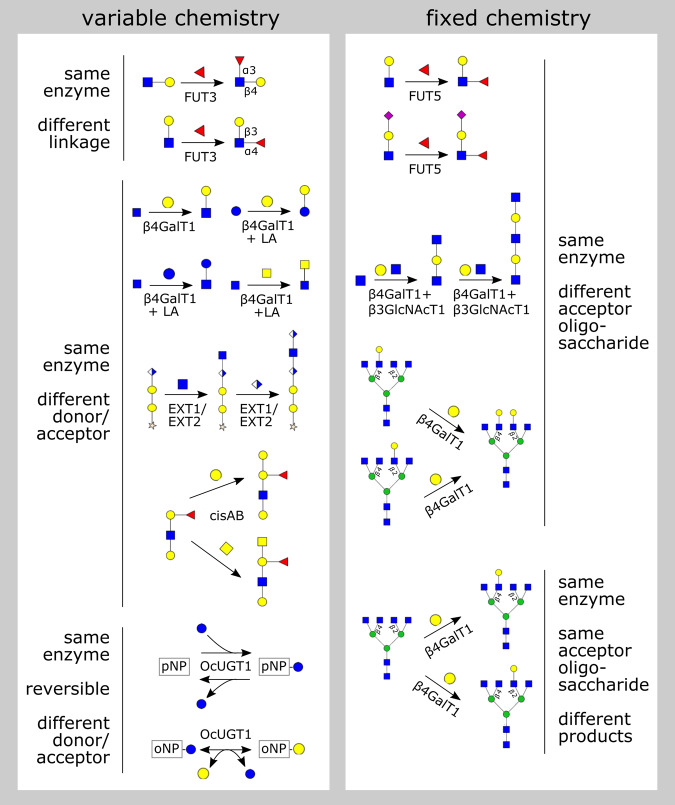
Classes of glycosyltransferase promiscuity. Each reaction (black arrow) is catalyzed by a specific enzyme (label on the arrow). We show the acceptor oligosaccharide on the left, the donor monosaccharide above or below the arrow (with the nucleotide omitted), and the product on the right. Reversible reactions are indicated with double arrows. See the text for details of each reaction.

New high-throughput experimental capabilities, including modular GTase expression systems [18], improved inference methods for determining enzymatic activity from mass spectrometry [11,19,20], and standardized glycan bioinformatic resources [21,22,23], are poised to yield comprehensive data on GTase substrate preferences. However, at present the most reliable data on GTases come from detailed studies of individual enzymes. While these classical studies span several decades, by collecting them together we can discern general principles governing the reaction patterns caused by GTase promiscuity (Figure 2). These principles will be essential to constrain computational models that aim to provide a predictive framework for protein and small-molecule glycosylation [11,12,24,25,26,27]. Here we discuss the causes and consequences promiscuity from a structural and biochemical perspective.

## Glycosyltransferase promiscuity

A promiscuous enzyme is one that catalyzes multiple reactions with distinct chemistries, or acts on multiple distinct substrates [28,29,30]. Promiscuity is determined by measuring the kinetics of enzymatic catalysis for each substrate/reaction combination. An enzyme will typically have a preferred or ‘canonical’ substrate and reaction, with a lower catalytic efficiency for ‘non-canonical’ substrates and reactions. In our discussion, we define the ‘chemistry’ of a glycosyltransferase reaction to mean the creation of a specific linkage between a specific donor monosaccharide and a specific acceptor small molecule or monosaccharide. We will first discuss examples of ‘variable-chemistry promiscuity’ in which the same GTase enzymes can catalyze multiple linkages or act on multiple donors or acceptors (Figure 2, left). We will then turn to the case of ‘fixed-chemistry promiscuity’, which is particularly relevant for understanding glycan biosynthesis (Figure 2, right).

## Variable-chemistry promiscuity in small-molecule synthesis

### Variable acceptors and donors, reversible reactions

GT-1 GTases involved in the synthesis of plant secondary metabolites (sometimes termed UDP glycosyltransferases or UGTs) represent an extreme limit of variable-chemistry promiscuity. A single UGT typically has multiple possible sugar donors and small-molecule acceptors [14,15,16,31,32]. Moreover, the reactions catalyzed by these enzymes are reversible under physiological conditions, meaning that the same enzyme can also act to cleave the sugar from the small molecule [31]. Taken together, this means that a single enzyme can perform nucleotide-sugar synthesis, transfer a sugar between different acceptors, or exchange different sugars on a single acceptor (Figure 2, left). For example, the enzyme OcUGT1 from the plant Ornithogalum caudatum can reversibly add or remove donor monosaccharaides such as glucose or galactose from dozens of acceptors [31].

## Variable-chemistry promiscuity in glycan synthesis

### Variable linkages

The fucosyltransferase enzymes FUT3–7 attach fucose in an α1–3-linkage to a Galβ1–4GlcNAc (LacNAc) acceptor. FUT3 and FUT5 can, in addition, attach fucose in an α1,4-linkage to a Galβ1–3GlcNAc acceptor [33,34,35,36,37]. This type of linkage promiscuity occurs during the biosynthesis of Lewis antigens (Figure 2, left).

### Variable acceptor and donor monosaccharaides

The canonical ‘galactosyltransferase’ activity of β-1,4-Galactosyltransferase-1 (β4GalT1) involves the attachment of a galactose donor to a GlcNAc acceptor [38]. However, the enzyme can also catalyze the attachment of other donors such as glucose and GalNAC to a GlcNAc acceptor, with an efficiency of 0.3% to 5% compared with the canonical activity [39]. The efficiency of these non-canonical reactions is enhanced by up to 30-fold when β4GalT1 forms a complex with the calcium-binding protein α-lactalbumin (LA) [38,39,40]. The β4GalT1-LA complex also displays ‘lactose synthase’ activity, via the attachment of the canonical galactose donor to a non-canonical glucose acceptor [38]. The activity of β4GalT1 requires an ‘open’ to ‘closed’ transition of its active site loop; LA binds to and stabilizes the enzyme's ‘closed’ conformation, enhancing its affinity for non-canonical substrates [41], thus making β4GalT1 more promiscuous.

Mammalian extostosin (EXT) proteins are GTases involved in heparan sulfate elongation [42]. The proteins EXT1 and EXT2 are promiscuous, each capable of both GlcA transferase activity and GlcNAc transferase activity, albeit at low efficiency [42,43]. The efficiency of these reactions is greatly amplified when EXT1 and EXT2 form a heterodimer [44]. The repeated addition of GlcA and GlcNAc generates a runaway reaction, leading to microheterogeneity in heparin sulfate tandem-repeat length (Figure 1B).

The synthesis of blood group antigens in humans is typically carried out by two different alleles of the ABO gene, GTA and GTB, that have distinct donors though they differ by just four critical residues [45,46]. The GTA allele converts the H-antigen to an A-antigen by attaching a GalNAc donor; the GTB allele converts the H-antigen to a B-antigen by attaching a galactose donor. However, the enzyme encoded by the cis-AB allele of the ABO gene (which is rare in humans but very common in mice) is promiscuous: it can use either a GalNAc donor or a galactose donor, to produce either the A-antigen or the B-antigen [47]. This is an example of microheterogeneity caused by a divergent reaction (Figure 1B).

## Fixed-chemistry promiscuity in glycan synthesis

During glycan synthesis, the acceptor substrates are complex oligosaccharides which potentially contain many sites to which a donor monosaccharide may be linked. This means that even if a GTase enzyme is specialized to catalyze reactions with fixed chemistry (a specific linkage between a specific donor and acceptor monosaccharide), it may still act on a variety of acceptor oligosaccharides, or generate a variety of products (Figure 2, right).

### Variable acceptor oligosaccharides

In the Lewis antigen biosynthetic pathway, FUT5 can attach fucose in an α1–3-linkage to both LacNAc and to Sia-ɑ2,3Galβ1–4GlcNAc acceptors [8, Chapters 48,50]. Thus FUT5 can act on acceptor oligosaccharides of varying sizes and compositions. The enzymes β4GalT1 (and the related β4GalT2) [48] can act on multiple distinct monogalactosylated oligosaccharides to produce a digalactosylated product. Thus these enzymes can thus act on acceptor oligosaccharides of varying structure.

An interesting case of variable-acceptor promiscuity is when a GTase can repeatedly act on acceptor oligosaccharides of increasing size. This contributes to runaway reactions, resulting in tandem-repeat microheterogeneity (Figure 1B). For example, poly-LacNAc repeats are found in complex multi antennary N-glycans, on i blood group antigens, and mucin glycoproteins [1, Chapter 14]. Poly-LacNAc is formed by the sequential action of the enzymes β4GalT1 and β-1,3GlcNAcT1 (β3GlcNAcT1), which increases the size of the chain by one LacNAc unit. Similarly, xylose-GlcA repeats are necessary for the proper functioning of α-dystroglycan, a glycoprotein involved in extracellular matrix integrity. These repeats are generated by LARGE-family GTases [49,50,51]: the proteins LARGE1 and LARGE2 each contain two fixed-chemistry catalytic domains, separately responsible for xylosyltransferase and glucuronyltransferase activities [8, Chapter 104].

### Same acceptor oligosaccharide, variable products

The enzyme β4GalT1 discussed above can convert the same ungalactosylated tetra-antennary N-glycan oligosaccharide to multiple distinct monogalactosylated products [48]. The substrate oligosaccharide has four GlcNAc termini on which β4GalT1 can act. Though the enzyme displays a hierarchy of preferences across these terminal monosaccharides, it can stochastically act on any of them to yield a monogalactosylated product. Three of the four monogalactosylated forms have been observed in an *in vitro* assay of enzyme activity [48]; it is likely that the fourth monogalactosylated form is rapidly converted to a digalactosylated form and is therefore not observed. The same enzyme can subsequently convert the monogalactosylated form into multi-galactosylated forms, causing the reaction paths to reconverge. However, this type of promiscuity can sill contribute to microheterogeneity if reactions are truncated due to early exit from the compartment (Figure 1B).

## Recognition motifs explain variations in GTase promiscuity

**Figure 3.**
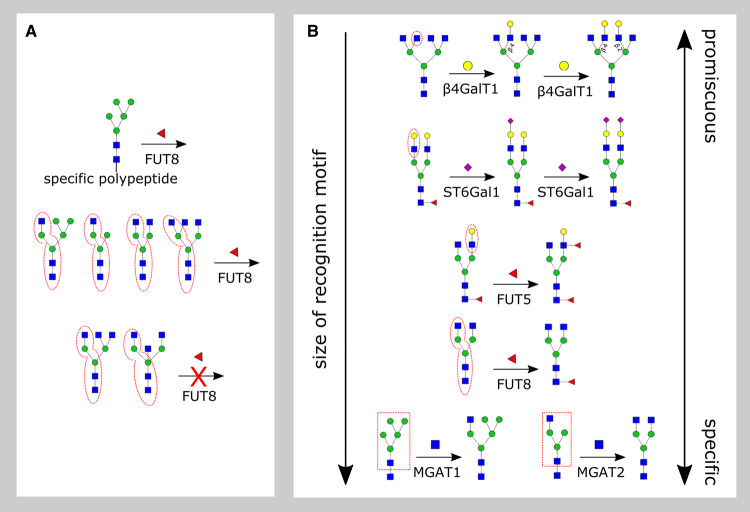
Glycosyltransferase recognition motifs. (**A**) To determine an enzyme's recognition motif, we require comprehensive data on all possible acceptor substrates on which it acts. Here we show the example of FUT8, which typically acts on any oligosaccharide acceptor containing its preferred motif (dashed red ovals). However, the enzyme can sometimes act when the motif is missing (top row) or fail to act when the motif is present (bottom row). (**B**) For each enzyme, we highlight its approximate recognition motif by dashed red ovals or boxes. Enzymes with small recognition motifs are highly promiscuous, those with large recognition motifs are highly specific.

There is often a highly specific, ‘necessary and sufficient’ acceptor substrate on which a given enzyme will show measurable activity (Figure 3). Consistent with this, crystal structures of substrate-bound GTases [6] suggest that enzymes make contacts with several moieties of the acceptor oligosaccharide beyond the specific donor and acceptor monosaccharides found in the active-site pocket. We term such a necessary and sufficient substrate the enzyme's ‘recognition motif’.

The determination of an enzyme's precise recognition motif is complex, involving detailed kinetic studies on a variety of potential acceptor substrates. Recent studies of the enzyme FUT8 [52,53,54] (discussed below) suggest that the concept of a recognition motif must be understood as approximate (Figure 3A). FUT8 acts on certain oligosaccharides that do not contain its canonical recognition motif, as long as they are linked to specific peptides; conversely, it does not act on certain oligosaccharides that contain extended forms of its recognition motif [55,56].

A GTase with a highly specific recognition motif can nevertheless be promiscuous. This is because recognition motifs often consist of just a few monosaccharaides, and the enzyme will typically act on any oligosaccharide which contains its recognition motif as a sub-structure. The smaller the recognition motif, the more oligosaccharides on which it will be found, and the more promiscuous the associated GTase will be. We now discuss several examples to illustrate the range of recognition motif sizes across different classes of GTases involved in glycan synthesis (Figure 3).

### Monosaccharide recognition

The β4GalT1 enzyme can act directly on a GlcNAc monosaccharide [8, Chapter 5] [57]. This enzyme is highly promiscuous, able to act in almost any context where this recognition motif is found on larger oligosaccharides (for example, on poly-LacNAc and tetra-antennary oligosaccharides discussed earlier).

### Disaccharide recognition

The fucosyltransferase enzyme FUT5 can act directly on the LacNAc disaccharide, attaching fucose to the GlcNAc monosaccharide contained within it [57]. During Lewis antigen biosynthesis, members of the FUT3–7 family of enzymes are known to act on multiple oligosaccharides containing this recognition motif [33]. Enzymes of the ST6Gal and ST3Gal sialyltransferases families attach sialic acid to the terminal galactose monosaccharides of both N-linked and O-linked glycans [58]. The recognition motifs of these enzymes are all variants of the LacNAc disaccharide. ST6Gal1 has LacNAc as its recognition motif [59], though it is observed to have a higher preference for the LacNAc at the α1,3-mannose arm of N-linked glycans [60]. ST6Gal2 has GalNAcβ1–4GlcNAc as its recognition motif, with LacNAc being an additional moiety on which it shows three-fold lower activity [61]. ST3Gal1 and ST3Gal2 both have Galβ1–3GalNAc as their recognition motifs, while ST3Gal3,4,6 show comparable activity on both LacNAc and Galβ1–3GlcNAc disaccharide moieties [62].

### Oligosaccharide recognition

The fucosyltransferase FUT8 attaches fucose to the Asn-linked root GlcNAc of N-glycans. This enzyme usually requires a β-linkage between the GlcNAc and the Asn, as well as a GlcNAc on the α1,3-mannose arm of the N-glycan core [55,56,63]. This is a large recognition motif which occurs on a restricted set of oligosaccharides. The requirement for this recognition motif may be dependent on the dimerization of FUT8, which allows its SH3 domain to form a specific binding pocket [64,65]. FUT8's activity is inhibited by the addition of a bisecting GlcNAc to its recognition motif [53,56], likely due to steric hindrance [64,65] (Figure 3A, bottom). However, FUT8 can fucosylate certain oligosaccharides lacking the α1,3-Man GlcNAc, if they are attached to specific peptides [52,54] (Figure 3A, top).

### Highly specific enzymes in N-glycan synthesis

Several enzymes act in a stereotypical, highly specific manner to modify N-glycan core oligosaccharides. For example, the enzyme MGAT1 efficiently attaches GlcNAc to the terminal ɑ1,3-mannose in Man_5_GlcNAc_2_ [1, Chapter 9], and shows a much lower affinity for di-mannose or tri-mannose variants of this oligosaccharide [66]. The enzymes MGAT3–4 specifically act on the product of MGAT1 [67]. The enzyme MGAT2 acts further along the pathway, attaching GlcNAc to the α1–6-mannose of GlcNAcMan_3_GlcNAc_2_ to generate the precursor of complex N-glycans [8, Chapter 18]. MGAT2 makes several specific interactions with the GlcNAc-β1,2Man-α1,3Manβ branch of its acceptor oligosaccharide [67]. Kinetic assays demonstrate that this recognition motif is essential for efficient MGAT2 activity [68] (the same motif is also recognized by MGAT3–4 [67]). The enzyme MGAT5 acts on the product of MGAT2, attaching a second GlcNAc to the α1–6-mannose. The crystal structure of MGAT5 shows the α1–6-mannose branch of its substrate buried within a deep cavity of the enzyme [69], contributing to its high degree of specificity.

## Discussion

GTases are versatile enzymes which play crucial roles in cellular physiology, catalyzing a large variety of sugar-addition reactions on many types of organic substrates. Though GTases are drawn from a small number of structural classes, they span the full spectrum of substrate preferences. The GT1 GTases that glycosylate small molecules represent the limit of extreme promiscuity, accommodating diverse donors and acceptors and catalyzing sugar addition as well as removal. In contrast, the GTases that build the N-glycan core represent the limit of extreme specificity: these enzymes each essentially act on a single acceptor oligosaccharide under physiological conditions.

## Perspectives

- Importance to the field. The study of glycosylation is increasingly relevant to understanding a variety of cell-biological processes. The majority eukaryotic cell-surface proteins are glycosylated, glycans influence inter-cellular interactions, and errors in glycan synthesis are implicated in several disorders. The precise origins of glycan variations between proteins, cell types and species remain open questions. Moreover, the synthesis of glycosylated proteins and small molecules has important applications in the production of biologic drugs and flavonoid-based nutritional supplements. The precise control of glycosylation at industrial scales remains a key challenge. A comprehensive understanding of glycosyltransferase promiscuity and specificity will be central to addressing these questions.
- Summary of current thinking. The field of eukaryotic glycobiology is split into two broad areas: first, the biochemistry glycosyltransferase enzymes; second, the cell biology of glycan synthesis, particularly Golgi biogenesis and the regulation of enzyme localization within Golgi compartments. It is increasingly clear that these two aspects of the field are tightly linked. The compartmentalized structure of the Golgi is likely to be a control mechanism that allows the tuning of glycan biosynthesis and the mitigation of variability caused by enzyme promiscuity.
- Future directions. The study of glycosyltransferase enzymes and glycosylated molecules is still a technically challenging field. As new analytic tools become available for quantitative glycomics, they will begin to generate rich data in a manner similar to what has already occurred for proteomics and lipidomics. A central challenge will be to develop approaches to manage, process, and extract meaning from such rich datasets, especially in a manner that connects to cell and organismal physiology. The development of new statistical approaches for chemical analytics, and of predictive computational models of glycan synthesis by GTases in the Golgi apparatus, represent an exciting interdisciplinary frontier for the field.

## Abbreviations

β3GlcNAcT1: β-1,3GlcNAcT1
β4GalT1: β-1,4-Galactosyltransferase-1
GalNAc: N-acetylgalactosamine
GlcNAc: N-acetylglucosamine
GlcA: glucuronic acid
GTA: α(1–3)-N-acetylgalactosaminyltransferase
GTase: glycosyltransferase
GTB: α(1–3)-galactosyltransferase
LA: α-lactalbumin
LacNAc: Galβ1–4GlcNAc
UDP: uridine diphosphate
